# 2-Phenyl-5-(*p*-tol­yl)-1,3,4-oxadiazole

**DOI:** 10.1107/S1600536811023579

**Published:** 2011-06-22

**Authors:** David B. Cordes, Guoxiong Hua, Alexandra M. Z. Slawin, J. Derek Woollins

**Affiliations:** a School of Chemistry, University of St Andrews, Fife KY16 9ST, Scotland

## Abstract

The title compound, C_15_H_12_N_2_O, adopts the expected near-planar geometry, the phenyl and tolyl rings being inclined relative to the oxadiazole ring by 3.8 (3) and 8.3 (2)°, respectively. This allows adjacent mol­ecules to pack in a parallel fashion and form stacking along [010] *via* π–π inter­actions [centroid–centroid distances = 3.629 (2) and 3.723 (2) Å]. Further inter­molecular inter­actions include C—H⋯π inter­actions and weak C—H⋯N hydrogen bonds, giving rise to a crossed herringbone packing motif.

## Related literature

For synthesis of the starting material *N*′-benzoyl-4-methyl­benzohydrazide, see: Hua *et al.* (2009 ▶). For a review of synthetic routes to the title compound, see: Weaver (2004 ▶). For related structures, see: Kuznetsov *et al.* (1998 ▶); Franco *et al.* (2003 ▶); Reck *et al.* (2003 ▶).
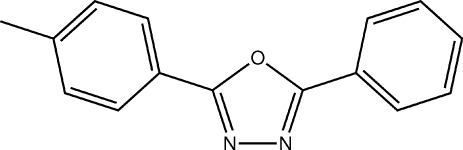


## Experimental

### 

#### Crystal data

C_15_H_12_N_2_O
*M* *_r_* = 236.27
Monoclinic, 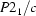

*a* = 19.733 (5) Å
*b* = 5.1441 (12) Å
*c* = 12.436 (3) Å
β = 107.477 (6)°
*V* = 1204.1 (5) Å^3^
*Z* = 4
Mo *K*α radiation
μ = 0.08 mm^−1^
*T* = 93 K
0.20 × 0.04 × 0.02 mm

#### Data collection

Rigaku Mercury CCD diffractometer
Absorption correction: multi-scan (*CrystalClear*; Rigaku, 2010 ▶) *T*_min_ = 0.984, *T*_max_ = 0.998
7407 measured reflections
2256 independent reflections
1293 reflections with *I* > 2σ(*I*)
*R*_int_ = 0.207

#### Refinement

*R*[*F* ^2^ > 2σ(*F* ^2^)] = 0.109
*wR*(*F* ^2^) = 0.307
*S* = 1.02
2256 reflections
164 parameters
H-atom parameters constrained
Δρ_max_ = 0.85 e Å^−3^
Δρ_min_ = −0.48 e Å^−3^

### 

Data collection: *CrystalClear* (Rigaku, 2010 ▶); cell refinement: *CrystalClear*; data reduction: *CrystalClear*; program(s) used to solve structure: *SHELXS97* (Sheldrick, 2008 ▶); program(s) used to refine structure: *SHELXL97* (Sheldrick, 2008 ▶); molecular graphics: *SHELXTL* (Sheldrick, 2008 ▶); software used to prepare material for publication: *SHELXTL* and *PLATON* (Spek, 2009 ▶).

## Supplementary Material

Crystal structure: contains datablock(s) global, I. DOI: 10.1107/S1600536811023579/su2281sup1.cif

Structure factors: contains datablock(s) I. DOI: 10.1107/S1600536811023579/su2281Isup2.hkl

Supplementary material file. DOI: 10.1107/S1600536811023579/su2281Isup3.cml

Additional supplementary materials:  crystallographic information; 3D view; checkCIF report

## Figures and Tables

**Table 1:** Hydrogen-bond geometry (Å, °) *Cg*1 and *Cg*2 are the centroids of the C3–C8 and C10–C15 rings, respectively.

*D*—H⋯*A*	*D*—H	H⋯*A*	*D*⋯*A*	*D*—H⋯*A*
C11—H11⋯N2^i^	0.95	2.61	3.322 (4)	132
C9—H9*B*⋯*Cg*1^ii^	0.98	2.80	3.731 (4)	158
C14—H14⋯*Cg*2^iii^	0.95	2.99	3.783 (4)	141

Symmetry codes: (i) 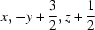; (ii) 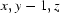; (iii) 
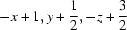
.

**Fig. 1.**
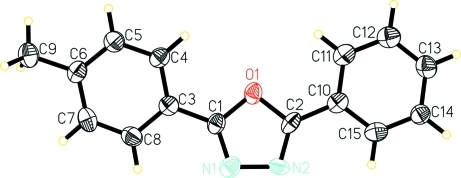
The molecular structure of the title compound with displacement ellipsoids drawn at the 50% probability level.

**Fig. 2.**
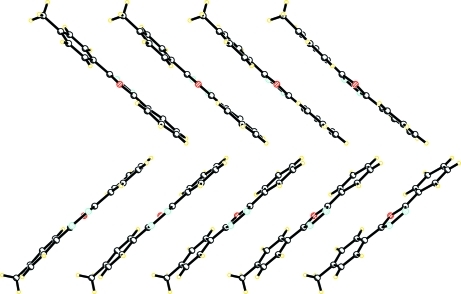
View down the c-axis showing the π-stacking of adjacent molecules and the formation of the herringbone sheet.

**Fig. 3.**
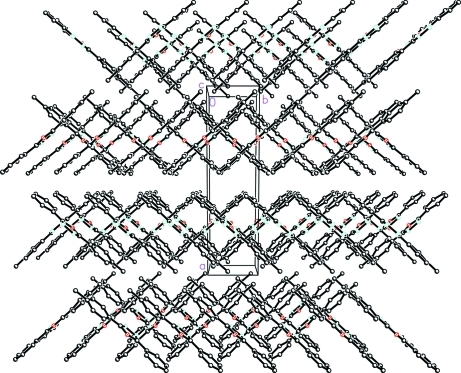
Packing diagram of the title compound showing the crossed herringbone pattern arising from offset of adjacent herringbone sheets. Hydrogen atoms were omitted for clarity.

## supplementary crystallographic information

### Comment

The title compound (Fig. 1), previously prepared by a number of different routes (Weaver, 2004), has been prepared by a new method, reacting Woollins' reagent with *N*'-benzoyl-4-methylbenzohydrazide (Hua *et al.*, 2009). It adopts an offset π-stacked packing motif, similar to those seen in related structures (Kuznetsov *et al.*, 1998, Franco *et al.*, 2003, and Reck *et al.*, 2003), the oxadiazole ring interacting with both the tolyl (*x*, 1 + *y*, *z*) and the phenyl (*x*, -1 + *y*, *z*) rings of adjacent molecules [centroid-centroid distances of 3.629 (2) and 3.723 (2) Å, respectively]. As a result of this arrangement, one of the tolyl methyl H atoms forms a C—H···π interaction with the adjacent tolyl π-system (Table 1). The stacks run along the [0 1 0] direction, and form herringbone sheets in the (0 0 1) plane (Fig. 2), *via* further C—H···π interactions (Table 1). These sheets resemble the herringbone packing motif seen previously in the structures of 2,5-diphenyl-1,3,4-oxadiazole (Kuznetsov *et al.*, 1998, and Franco *et al.*, 2003). However, adjacent sheets do not align, and instead are offset, forming a crossed herringbone pattern (Fig. 3), interacting *via* C—H···N hydrogen bonds (Table 1).

Footnote to Table 1: Cg1 = centroid of ring (C3-C8); Cg2 = centroid of ring (C10-C15).

### Experimental

A red suspension of *N*'-benzoyl-4-methylbenzohydrazide (0.25 g, 1.0 mmol, Hua *et al.*, 2009) and Woollins' reagent (0.54 g, 1.0 mmol) in 20 ml of dry toluene was refluxed for 7 h. Following cooling to room temperature and removal of the solvent *in vacuuo* the residue was purified by silica gel column chromatography (1: 9 ethyl acetate/dichloromethane eluent) to give 2-phenyl-5-*p*-tolyl-1,3,4-selenadiazole as a dark yellow solid in good yield (0.270 g, 90%). The title compound was formed from this by air oxidation during the growth of X-ray quality crystals from the diffusion of hexane into a dichloromethane solution of 2-phenyl-5-*p*-tolyl-1,3,4-selenadiazole.

### Refinement

The crystal initially chosen appeared to be poorly diffracting at higher angles, so several others were also tried. All were found to be weakly diffracting, resulting in a number of missing independent data in the experimentally measured range. One low angle reflection (1 0 4) was omitted due to being partially behind the beamstop. All H atoms were included in calculated positions (C—H distances are 0.98 Å for methyl H atoms and 0.95 Å for phenyl H atoms) and refined as riding atoms with *U*_iso_(H) = 1.2 *U*_eq_(parent atom, phenyl H atoms) or *U*_iso_(H) = 1.5 *U*_eq_ (parent atom, methyl H atoms). The highest electron density peak is located 1.19 Å from atom O1.

### Crystal data

**Table:**

C_15_H_12_N_2_O	*F*(000) = 496
*M**_r_* = 236.27	*D*_x_ = 1.303 Mg m^−^^3^
Monoclinic, *P*2_1_/*c*	Mo *K*α radiation, λ = 0.71073 Å
Hall symbol: -P 2ybc	Cell parameters from 2748 reflections
*a* = 19.733 (5) Å	θ = 3.3–28.2°
*b* = 5.1441 (12) Å	µ = 0.08 mm^−^^1^
*c* = 12.436 (3) Å	*T* = 93 K
β = 107.477 (6)°	Chip, colourless
*V* = 1204.1 (5) Å^3^	0.20 × 0.04 × 0.02 mm
*Z* = 4	

### Data collection

**Table:**

Rigaku Mercury CCD diffractometer	2256 independent reflections
Radiation source: rotating anode	1293 reflections with *I* > 2σ(*I*)
confocal	*R*_int_ = 0.207
Detector resolution: 14.7059 pixels mm^-1^	θ_max_ = 27.5°, θ_min_ = 3.3°
ω and φ scans	*h* = −24→24
Absorption correction: multi-scan (*CrystalClear*; Rigaku, 2010)	*k* = −3→6
*T*_min_ = 0.984, *T*_max_ = 0.998	*l* = −11→14
7407 measured reflections	

### Refinement

**Table:**

Refinement on *F*^2^	Primary atom site location: structure-invariant direct methods
Least-squares matrix: full	Secondary atom site location: difference Fourier map
*R*[*F*^2^ > 2σ(*F*^2^)] = 0.109	Hydrogen site location: inferred from neighbouring sites
*wR*(*F*^2^) = 0.307	H-atom parameters constrained
*S* = 1.02	*w* = 1/[σ^2^(*F*_o_^2^) + (0.1532*P*)^2^] where *P* = (*F*_o_^2^ + 2*F*_c_^2^)/3
2256 reflections	(Δ/σ)_max_ < 0.001
164 parameters	Δρ_max_ = 0.85 e Å^−^^3^
0 restraints	Δρ_min_ = −0.48 e Å^−^^3^

### Special details

**Table:**

Geometry. All e.s.d.'s (except the e.s.d. in the dihedral angle between two l.s. planes) are estimated using the full covariance matrix. The cell e.s.d.'s are taken into account individually in the estimation of e.s.d.'s in distances, angles and torsion angles; correlations between e.s.d.'s in cell parameters are only used when they are defined by crystal symmetry. An approximate (isotropic) treatment of cell e.s.d.'s is used for estimating e.s.d.'s involving l.s. planes.
Refinement. Refinement of *F*^2^ against ALL reflections. The weighted *R*-factor *wR* and goodness of fit *S* are based on *F*^2^, conventional *R*-factors *R* are based on *F*, with *F* set to zero for negative *F*^2^. The threshold expression of *F*^2^ > σ(*F*^2^) is used only for calculating *R*-factors(gt) *etc*. and is not relevant to the choice of reflections for refinement. *R*-factors based on *F*^2^ are statistically about twice as large as those based on *F*, and *R*- factors based on ALL data will be even larger.

### Fractional atomic coordinates and isotropic or equivalent isotropic displacement parameters (Å^2^)

**Table:**

	*x*	*y*	*z*	*U*_iso_*/*U*_eq_	
O1	0.26886 (13)	0.5359 (5)	0.70037 (19)	0.0337 (8)	
N1	0.24323 (17)	0.4502 (6)	0.5190 (3)	0.0374 (9)	
N2	0.29187 (16)	0.6544 (6)	0.5435 (2)	0.0334 (9)	
C1	0.2307 (2)	0.3862 (7)	0.6126 (3)	0.0322 (10)	
C2	0.30560 (19)	0.6981 (7)	0.6502 (3)	0.0299 (10)	
C3	0.18313 (19)	0.1875 (8)	0.6309 (3)	0.0306 (10)	
C4	0.1822 (2)	0.1219 (7)	0.7391 (3)	0.0330 (10)	
H4	0.2132	0.2077	0.8027	0.040*	
C5	0.1364 (2)	−0.0672 (8)	0.7541 (3)	0.0354 (10)	
H5	0.1369	−0.1109	0.8285	0.042*	
C6	0.0898 (2)	−0.1957 (8)	0.6643 (3)	0.0344 (10)	
C7	0.0919 (2)	−0.1274 (8)	0.5552 (3)	0.0413 (11)	
H7	0.0606	−0.2120	0.4917	0.050*	
C8	0.1378 (2)	0.0573 (8)	0.5385 (3)	0.0374 (11)	
H8	0.1388	0.0967	0.4643	0.045*	
C9	0.0394 (2)	−0.3978 (8)	0.6791 (3)	0.0421 (11)	
H9A	0.0416	−0.4078	0.7588	0.063*	
H9B	0.0524	−0.5665	0.6544	0.063*	
H9C	−0.0090	−0.3520	0.6339	0.063*	
C10	0.35306 (19)	0.8918 (7)	0.7193 (3)	0.0307 (10)	
C11	0.3641 (2)	0.9055 (7)	0.8354 (3)	0.0368 (11)	
H11	0.3400	0.7895	0.8708	0.044*	
C12	0.4102 (2)	1.0888 (8)	0.8982 (3)	0.0400 (11)	
H12	0.4191	1.0945	0.9776	0.048*	
C13	0.4437 (2)	1.2640 (8)	0.8470 (3)	0.0392 (11)	
H13	0.4743	1.3924	0.8910	0.047*	
C14	0.4327 (2)	1.2533 (7)	0.7316 (3)	0.0347 (10)	
H14	0.4565	1.3710	0.6964	0.042*	
C15	0.3871 (2)	1.0705 (7)	0.6692 (3)	0.0344 (10)	
H15	0.3785	1.0657	0.5898	0.041*	

### Atomic displacement parameters (Å^2^)

**Table:**

	*U*^11^	*U*^22^	*U*^33^	*U*^12^	*U*^13^	*U*^23^
O1	0.0369 (16)	0.0242 (16)	0.0432 (18)	−0.0030 (12)	0.0167 (13)	−0.0033 (11)
N1	0.041 (2)	0.032 (2)	0.042 (2)	0.0020 (17)	0.0165 (16)	0.0037 (14)
N2	0.042 (2)	0.035 (2)	0.0250 (19)	−0.0011 (16)	0.0129 (14)	−0.0061 (13)
C1	0.034 (2)	0.023 (2)	0.040 (2)	0.0001 (18)	0.0135 (17)	0.0024 (17)
C2	0.034 (2)	0.019 (2)	0.044 (2)	0.0064 (17)	0.0223 (18)	0.0073 (16)
C3	0.032 (2)	0.027 (2)	0.036 (2)	0.0054 (17)	0.0139 (16)	−0.0013 (16)
C4	0.035 (2)	0.032 (2)	0.035 (2)	0.0026 (18)	0.0156 (17)	0.0019 (16)
C5	0.039 (2)	0.032 (2)	0.036 (2)	0.0031 (19)	0.0132 (18)	−0.0024 (17)
C6	0.037 (2)	0.026 (2)	0.045 (3)	0.0041 (18)	0.0200 (18)	0.0008 (17)
C7	0.040 (3)	0.031 (3)	0.050 (3)	−0.002 (2)	0.0088 (19)	−0.0091 (18)
C8	0.043 (3)	0.035 (3)	0.033 (2)	0.001 (2)	0.0103 (18)	−0.0036 (17)
C9	0.039 (2)	0.035 (3)	0.052 (3)	0.003 (2)	0.013 (2)	−0.0038 (18)
C10	0.035 (2)	0.022 (2)	0.040 (2)	0.0086 (17)	0.0188 (18)	−0.0012 (16)
C11	0.046 (3)	0.027 (2)	0.043 (3)	0.005 (2)	0.0228 (19)	0.0003 (17)
C12	0.051 (3)	0.033 (3)	0.040 (3)	0.005 (2)	0.017 (2)	−0.0025 (18)
C13	0.042 (3)	0.032 (3)	0.044 (3)	0.000 (2)	0.0139 (19)	−0.0099 (18)
C14	0.038 (2)	0.030 (2)	0.035 (2)	−0.0008 (19)	0.0098 (17)	0.0056 (17)
C15	0.040 (2)	0.033 (2)	0.030 (2)	0.0092 (19)	0.0103 (17)	0.0068 (17)

### Geometric parameters (Å, °)

**Table:**

O1—C1	1.363 (4)	C7—H7	0.9500
O1—C2	1.372 (4)	C8—H8	0.9500
N1—C1	1.304 (4)	C9—H9A	0.9800
N1—N2	1.393 (4)	C9—H9B	0.9800
N2—C2	1.293 (4)	C9—H9C	0.9800
C1—C3	1.451 (5)	C10—C15	1.391 (5)
C2—C10	1.458 (5)	C10—C11	1.396 (5)
C3—C4	1.394 (5)	C11—C12	1.379 (6)
C3—C8	1.397 (5)	C11—H11	0.9500
C4—C5	1.378 (5)	C12—C13	1.381 (5)
C4—H4	0.9500	C12—H12	0.9500
C5—C6	1.383 (5)	C13—C14	1.386 (5)
C5—H5	0.9500	C13—H13	0.9500
C6—C7	1.414 (5)	C14—C15	1.371 (5)
C6—C9	1.488 (5)	C14—H14	0.9500
C7—C8	1.371 (5)	C15—H15	0.9500
			
C1—O1—C2	102.7 (3)	C3—C8—H8	120.1
C1—N1—N2	107.3 (3)	C6—C9—H9A	109.5
C2—N2—N1	105.9 (3)	C6—C9—H9B	109.5
N1—C1—O1	111.4 (3)	H9A—C9—H9B	109.5
N1—C1—C3	128.5 (4)	C6—C9—H9C	109.5
O1—C1—C3	120.1 (3)	H9A—C9—H9C	109.5
N2—C2—O1	112.6 (3)	H9B—C9—H9C	109.5
N2—C2—C10	128.6 (3)	C15—C10—C11	118.9 (4)
O1—C2—C10	118.8 (3)	C15—C10—C2	119.9 (3)
C4—C3—C8	119.2 (4)	C11—C10—C2	121.1 (3)
C4—C3—C1	121.2 (4)	C12—C11—C10	119.5 (4)
C8—C3—C1	119.6 (3)	C12—C11—H11	120.3
C5—C4—C3	120.0 (4)	C10—C11—H11	120.3
C5—C4—H4	120.0	C11—C12—C13	120.7 (4)
C3—C4—H4	120.0	C11—C12—H12	119.6
C4—C5—C6	122.2 (3)	C13—C12—H12	119.6
C4—C5—H5	118.9	C12—C13—C14	120.3 (4)
C6—C5—H5	118.9	C12—C13—H13	119.9
C5—C6—C7	116.9 (4)	C14—C13—H13	119.9
C5—C6—C9	122.8 (3)	C15—C14—C13	119.0 (3)
C7—C6—C9	120.4 (4)	C15—C14—H14	120.5
C8—C7—C6	121.9 (4)	C13—C14—H14	120.5
C8—C7—H7	119.0	C14—C15—C10	121.5 (3)
C6—C7—H7	119.0	C14—C15—H15	119.2
C7—C8—C3	119.7 (3)	C10—C15—H15	119.2
C7—C8—H8	120.1		
			
C1—N1—N2—C2	0.4 (4)	C5—C6—C7—C8	0.0 (6)
N2—N1—C1—O1	−0.4 (4)	C9—C6—C7—C8	−179.8 (4)
N2—N1—C1—C3	179.0 (4)	C6—C7—C8—C3	−1.4 (6)
C2—O1—C1—N1	0.3 (4)	C4—C3—C8—C7	1.7 (6)
C2—O1—C1—C3	−179.3 (3)	C1—C3—C8—C7	−178.9 (3)
N1—N2—C2—O1	−0.2 (4)	N2—C2—C10—C15	4.1 (6)
N1—N2—C2—C10	−179.9 (4)	O1—C2—C10—C15	−175.5 (3)
C1—O1—C2—N2	0.0 (4)	N2—C2—C10—C11	−177.2 (4)
C1—O1—C2—C10	179.7 (3)	O1—C2—C10—C11	3.2 (5)
N1—C1—C3—C4	172.1 (4)	C15—C10—C11—C12	−2.3 (5)
O1—C1—C3—C4	−8.5 (5)	C2—C10—C11—C12	179.0 (3)
N1—C1—C3—C8	−7.3 (6)	C10—C11—C12—C13	2.2 (6)
O1—C1—C3—C8	172.1 (3)	C11—C12—C13—C14	−1.8 (6)
C8—C3—C4—C5	−0.7 (5)	C12—C13—C14—C15	1.5 (6)
C1—C3—C4—C5	179.9 (3)	C13—C14—C15—C10	−1.7 (6)
C3—C4—C5—C6	−0.7 (6)	C11—C10—C15—C14	2.1 (5)
C4—C5—C6—C7	1.1 (6)	C2—C10—C15—C14	−179.2 (3)
C4—C5—C6—C9	−179.1 (3)		

### Hydrogen-bond geometry (Å, °)

**Table:**

Cg1 and Cg2 are the centroids of the C3–C8 and C10–C15 rings, respectively.

**Table:**

*D*—H···*A*	*D*—H	H···*A*	*D*···*A*	*D*—H···*A*
C11—H11···N2^i^	0.95	2.61	3.322 (4)	132.
C9—H9B···Cg1^ii^	0.98	2.80	3.731 (4)	158.
C14—H14···Cg2^iii^	0.95	2.99	3.783 (4)	141.

Symmetry codes: (i) *x*, −*y*+3/2, *z*+1/2; (ii) *x*, *y*−1, *z*; (iii) −*x*+1, *y*+1/2, −*z*+3/2.
